# Wireless Charging System Using Resonant Inductor in Class E Power Amplifier for Electronics and Sensors

**DOI:** 10.3390/s20102801

**Published:** 2020-05-14

**Authors:** Feng Wen, Xingchen Cheng, Qiang Li, Jianqiao Ye

**Affiliations:** 1 School of Automation, Nanjing University of Science and Technology, Nanjing 210094, China; 118110022204@njust.edu.cn (X.C.); chnliqiang@njust.edu.cn (Q.L.); 18362902767@163.com (J.Y.); 2 Jiangsu Provincial Key Laboratory of Smart Grid Technology and Equipment, Nanjing 210096, China

**Keywords:** class E power amplifier, wireless power transfer (WPT), resonant inductor, electronics, sensors

## Abstract

This study aims to solve a problem that exists with impedance matching networks in terms of extra cost and power loss of electronic components in a four-coil wireless power transfer (WPT) system using class E power amplifier as power supply, which is not conducive to the improvement of system efficiency and output power. A design method of sharing the resonant inductor in class E power amplifier and the excitation coil in the four-coil WPT system is proposed. This method comprehensively considers the output power and transfer efficiency of the system, the number of coil turns, coil size and many other factors. Compared with the traditional four-coil system using a class E power amplifier as a power supply, the proposed method simplified the system structure by leaving out a resonant inductor and load matching circuit, which can reduce the power loss of system and improve efficiency. Moreover, the precisely tuning of resonant inductor was not necessary, which improved the stability of the system. The correctness and feasibility of the parameter design method were verified by experiments. The experimental results showed that the output power of the system was increased by 18.7%, the efficiency was increased by 11%, and the transmission distance was up to 0.7 m, which is suitable for wireless power supply of electronics and sensors.

## 1. Introduction

Since 2007, wireless power transfer (WPT) technologies have been widely concerned and studied because of their flexibility and security in power supply [1,2,3,4]. It is troublesome to use a wired charging mode to charge electronics and sensors spread across a geographical area. However, existing wireless charging equipment has a short transmission distance. Four-coil WPT systems with class E power amplifiers as high frequency power supply are widely researched because of the advantages of long transfer distance, simple structure and high efficiency. In reference [5], a novel four-resonator coil structure is presented to improve system efficiency. The source and load coils are designed to acquire high-loaded Q (quality factor) and maximum cross coupling coefficients, which increase system efficiency effectively. In reference [6], a high-efficiency WPT system with an asymmetric four-coil resonator is proposed. Two intermediate coils boost the apparent coupling coefficient at around the operating frequency. Reference [7] proposes methods to improve the transmission range by optimizing the magnetic mechanism and the distribution of auxiliary coils. Reference [8] demonstrates, explains and analyzes frequency-splitting phenomenon by using the circuit theory. A solution is proposed that helps improve the efficiency when frequency-splitting occurs. The theoretical calculations and experimental results provide a sound basis. In reference [9], a four-coil WPT system is designed by laying a lumped coil on the ground to realize the wireless power supply over a distance of 0.4 m. The transfer distance is not enough for modern wireless systems especially for electronics and sensors, since they may be widely distributed in high-voltage facilities, for example, the Ubiquitous Electric Internet of Things (UEIOT). The power transfer distance needs to be increased to ensure the safety of charging process. In reference [10], the equivalent circuit model of four coil wireless energy transmission system is established and the efficiency analysis is carried out. The expression of coupling coefficient between coils is derived when the maximum system efficiency is achieved. It can be applied to optimize the parameters of four-coil system. Reference [11] analyzes the relationship between transmission efficiency, transmission distance and mutual inductance when using four-coil system to charge laptop, and the transmission efficiency is up to 52%. Further improvement on the transfer efficiency is necessary in practical applications.

For the traditional four-coil WPT system, the impedance matching network to make the equivalent resistance of coil system consistent with the ideal load of class E power supply is essential to keep high performance of output power. However, the impedance matching network will inevitably increase the system complexity and results in extra power loss. The existing studies mainly concentrate on improving efficiency and transmission distance of four-coil WPT system by optimizing the coupling coils, however, the solutions of integrated design of power supply topology and four coil coupling mechanism are not intensively researched.

In this study, we propose a high-performance medium and long-distance wireless charging system with four-coil WPT system and class E power amplifier sharing a single resonant inductor. The proposed system does not use additional impedance matching network which reduces the complexity of the traditional four-coil system and has the characteristics of high efficiency, flexibility and long transmission distance. First, the theoretical model of four-coil system using resonant inductor in class E power amplifier is established and the principle of the model is analyzed in detail. The coil system is modeled based on mutual inductance theory and analyzed as the equivalent input resistance of class E power amplifier. The equivalent input resistance can be adjusted by the design of coil radius, turns and distances between coils. Second, the output voltage of class E power amplifier can be described as a function of input resistance when other parameters are constant. The design of power supply and four-coil system are combined for consideration. Under the given design requirements, the output power and efficiency of the system can be expressed as a function of turns and diameter of coils only. Finally, an experimental prototype is designed based on this method and verified by simulation and experimental results. The performance of the traditional four-coil WPT system and the proposed WPT system using resonant inductor in class E power amplifier are compared. The experimental results show that the output power of the system is increased by 18.7%, the efficiency is increased by 11% and the transmission distance is up to 0.7 m, which is suitable for wireless power supply of electronics and sensors.

## 2. Principle and Theoretical Model

The resonant frequency of the LC resonant circuit in class E power amplifier is the same as that of four-coil WPT system. As a result, the resonant inductor L in class E power amplifier can be used as excitation coil in four-coil WPT system. In this theoretical model, it is necessary to design parameters of excitation coil in four-coil system to make its inductance consistent with that of resonant inductance in class E power amplifier. In addition, it is necessary to design parameters of four coils to make the equivalent input resistance of four-coil system match the optimal load resistance of class E power amplifier. In this way, class E power amplifier can leave out the impedance matching network, so as to simplify the system structure and reduce the power loss.

**Figure 1:**
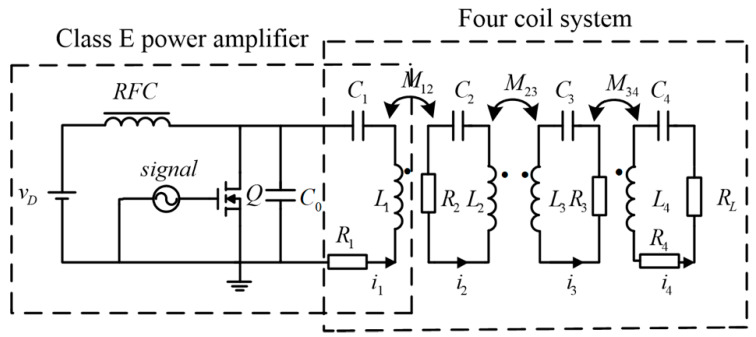
Circuit schematic of the proposed wireless power transfer (WPT) system.

Figure 1 shows the circuit schematic of the proposed WPT system using resonant inductor in class E power amplifier. In this system, SS (SS is a basic circuit topology in WPT system, which is characterized by the fact that transmitter coil and its tuning capacitor are in series resonance, receiver coil and its tuning capacitor are in series resonance) compensation structure is adopted for simplification. The circuit includes DC input power supply *V*_D_, gate drive signal, switch *Q*, capacitor in parallel *C*_0_, excitation coil and resonant inductor *L*_1_, transmitter coil *L*_2_, receiver coil *L*_3_, load coil *L*_4_, load *R_L_* of four-coil system, resonant capacitor *C*_1_, *C*_2_, *C*_3_, *C*_4_ and parasitic resistance *R*_1_, *R*_2_, *R*_3_, *R*_4_. Residual inductor of traditional class E power amplifier which provides a certain inductive reactance for the circuit to improve the power factor is not concerned in this study since we focus on streamlined WPT structure and excellent transfer efficiency.

First of all, the ideal working conditions of class E power amplifier is analyzed.

**Figure 2:**
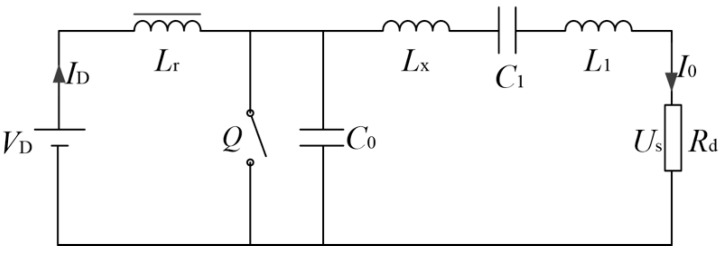
Equivalent circuit of class E power amplifier.

Figure 2 is the equivalent circuit of class E power amplifier. *L_r_* is the high frequency choke, *L_X_* is the residual inductor, *R_d_* is the load resistance, *I*_0_ and *U_s_* is the load current and output voltage. The relationship between the load resistance and the output voltage of class E power amplifier is given in reference [12]. When the duty cycle of the driving signal is 0.5, we can get:

(1) $$\tan\varphi =\frac{\pi^{2}-2\pi^{2}\omega C_{0}X-4\pi \omega C_{0}R_{d}-8}{\pi \left(2\pi \omega C_{0}R_{d}-4\omega C_{0}X\right)+1}$$

(2) $$I_{D}=4\pi \omega C_{0}V_{D}\frac{\pi \omega C_{0}R_{d}+2\cos^{2}\varphi }{\pi^{3}\omega C_{0}R_{d}+8\sin^{2}\varphi }$$

(3) $$I_{0}=\frac{\pi \cos\varphi -2\sin\varphi }{\pi \omega C_{0}R_{d}+2\cos^{2}\varphi }I_{D}$$

(4) $$U_{s}=\frac{1}{\sqrt{2}}I_{0}R_{d}$$

(5) $$P_{0}=U_{s}I_{0}$$

where *X* = *ω L*_1_ − 1/( *ω C*_1_), *ω* is the angular frequency, *φ* is the phase of load current, *I_D_* is the DC input current, *P*_0_ is the output power. According to Formula (1), in class E power amplifier, when the parameters except the load resistance are determined, the load resistance has little effect on the phase (This can also be verified in Figure 5a in Section 3.2). Hence, the phase can be approximately considered as constant. According to Formula (2)–(4), when the phase is considered constant, output voltage *U_s_* can be regarded as related only to the load resistance.

The transfer efficiency [13] of class E power amplifier is also a key index of system performance that can be expressed by:

(6) $$\eta_{{}_{E}}=\frac{1}{1+\frac{(\pi^{2}+28)r_{DS}}{2(\pi^{2}+4)R_{d}}+\frac{P_{ton}}{P_{0}}+\frac{r_{L}}{R_{d}}+\frac{{\left(\omega t_{f}\right)}^{2}}{12}}$$

(7) $$P_{ton}=\frac{1}{2}fC_{0}V_{\mathrm{on}}{}^{2}=\frac{{\left(\pi I_{D}-2I_{0}\cos\varphi \right)}^{2}}{4\pi C_{0}\omega }$$

where, *r_DS_* is the on resistance of switch, *P_ton_* is the turn-on loss, *r_L_* is the parasitic resistance of resonant inductor, *t_f_* is the fall time of switching signal, *f* is the power frequency which equals 2*π*/*ω*. It can be seen from above analysis that the main power loss comes from switching loss. Therefore, to obtain an efficient class E power amplifier, a switch with low on resistance and short on-off time is essential.

According to Formula (1)–(7), the power and efficiency of class E power amplifier are obtained. For a class E power amplifier with a duty cycle of 0.5, the ideal optimal working conditions [14] can be obtained by:

(8) $$R=\frac{8}{\pi^{2}+4}\frac{V_{D}{}^{2}}{P_{0}}$$

(9) $$L_{1}=\frac{Q_{L}}{2\pi f}R$$

(10) $$C_{1}=\frac{1}{2\pi fR(Q_{L}-1.1525)}$$

(11) $$C_{0}=\frac{8}{\pi (\pi^{2}+4)2\pi fR}$$

where, *R* is the ideal load resistance of class E amplifier when duty cycle is 0.5, *Q_L_* is the quality factor of the excitation coil.

According to the circuit schematic shown in Figure 1, the Kirchhoff laws (KVL) equation of four-coil system using resonant inductor in class E power amplifier can be listed:

(12) $$\left[\begin{array}{cccc} 0 & j\omega M_{12} & 0 & 0\\ j\omega M_{12} & Z_{2} & j\omega M_{23} & 0\\ 0 & j\omega M_{23} & Z_{3} & j\omega M_{34}\\ 0 & 0 & j\omega M_{34} & Z_{4}+R_{L} \end{array}\right]\left[\begin{array}{c} \overset{•}{I_{1}}\\ \overset{•}{I_{2}}\\ \overset{•}{I_{3}}\\ \overset{•}{I_{4}} \end{array}\right]=\left[\begin{array}{c} \overset{•}{U_{s}}\\ 0\\ 0\\ 0 \end{array}\right]$$

where, *M*_12_ is the mutual inductance between excitation and transmitter coil, *M*_23_ is the mutual inductance between transmitter and receiver coil, *M*_34_ is the mutual inductance between receiver and load coil. *Z*_2_ *= R*_2_ *+* *jωL*_2_ *+* 1*/jωC*_2_, *Z*_3_ *= R*_3_ *+* *jωL*_3_ *+* 1*/jωC*_3_, *Z*_4_ *= R*_4_ *+* *jωL*_4_ *+* 1*/jωC*_4_. The mutual inductance between coils far apart is ignored.

For the purpose of achieving high transmission efficiency, all coils are compensated to resonance to reduce the reactive loss except the excitation one. Therefore, *Im_(_Z*_2*)*_ *= Im_(_Z*_3*)*_ *= Im_(_Z*_4*)*_ *=* 0. Since excitation coil is the resonant inductor in class E power amplifier, its value can be obtained in Formula (9) and (10) with capacitor *C*_1_.

According to Formula (12), the output power, the transfer efficiency and the equivalent input resistance of the four coils can be obtained:

(13) $$\left\{ \begin{array}{l} P_{oc}=\left|{\overset{•}{I}}_{4}^{2}R_{L}\right|=\\ \frac{M_{12}{}^{2}M_{23}{}^{2}M_{34}{}^{2}R_{L}Us^{2}\omega^{6}}{{\left(M_{12}{}^{2}R_{3}R_{4}\omega^{2}+M_{12}{}^{2}R_{3}R_{L}\omega^{2}+M_{12}{}^{2}M_{34}{}^{2}\omega^{4}\right)}^{2}}\\ \eta_{c}=\frac{P_{o}}{\overset{•}{I_{1}}\overset{•}{U_{S}}}=M_{12}{}^{2}M_{23}{}^{2}M_{34}{}^{2}R_{L}\omega^{6}/\\ {}[\left(M_{12}{}^{2}R_{3}R_{4}\omega^{2}+M_{12}{}^{2}R_{3}R_{L}\omega^{2}+M_{12}{}^{2}M_{34}{}^{2}\omega^{4}\right)\\ \left(R_{2}R_{3}R_{4}+R_{2}R_{3}R_{L}+M_{34}{}^{2}R_{2}\omega^{2}+M_{23}{}^{2}R_{4}\omega^{2}+M_{23}{}^{2}R_{L}\omega^{2}\right)]\\ R_{in}=M_{12}{}^{2}\omega^{2}/(R_{2}+M_{23}{}^{2}\omega^{2}/(R_{3}+M_{34}{}^{2}\omega^{2}/(R_{4}+R_{L})))=\\ \frac{M_{12}{}^{2}\omega^{2}\left(R_{3}R_{4}+R_{3}R_{L}+M_{34}{}^{2}\omega^{2}\right)}{R_{2}R_{3}R_{4}+R_{2}R_{3}R_{L}+M_{34}{}^{2}R_{2}\omega^{2}+M_{23}{}^{2}R_{4}\omega^{2}+M_{23}{}^{2}R_{L}\omega^{2}} \end{array}\right.$$

where, *P_OC_* is the output power of the proposed WPT system using resonant inductor in class E power amplifier. *η_c_* is the efficiency of the system. *R_in_* is the equivalent input resistance of the four coils.

**Figure 3:**
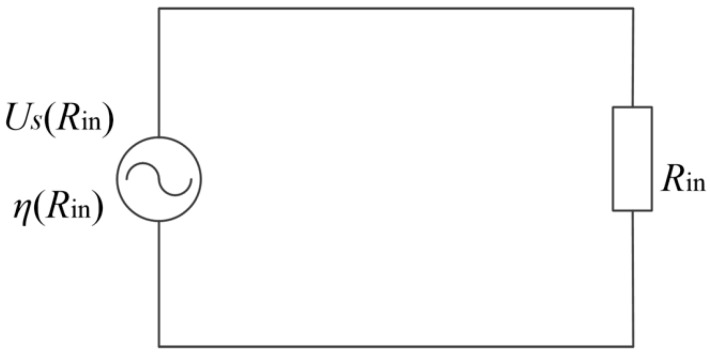
Simplified equivalent circuit of the proposed WPT system.

According to Formula (13), the proposed WPT system using resonant inductor in class E power amplifier can be equivalent to a series structure of power supply voltage and input resistance. The schematic diagram of equivalent circuit is shown in Figure 3. Where, *U_s_* (*R_in_*) is the output voltage of class E power amplifier when the equivalent input resistance is equal to *R_in_*. *η* (*R_in_*) is the efficiency of class E power amplifier when the equivalent input resistance is equal to *R_in_*. It can be noted that the target of obtaining high output power and efficiency is transformed to figure out the optimized values of mutual inductance between coils and equivalent input resistance.

The coils involved in this study are coaxial spiral coils, which are wound with copper wire and with turn spacing to reduce the influence of proximity effect. The calculation formula of parasitic resistance of spiral coil [15] is as follows:

(14) $$R=\sqrt{\frac{\omega \mu_{0}}{2\sigma }}\frac{Nr}{a}$$

where, *μ*_0_ is permeability of vacuum, *σ* is the conductivity, *a* is the copper wire radius, *N* is the turns of coil, *r* is coil radius.

Mutual inductance between coils can be obtained by Neumann Formula [16]:

(15) $$M=\frac{N_{1}N_{2}\mu_{0}}{4\pi }\int_{0}^{2\pi }d\phi \int_{0}^{2\pi }\frac{r_{1}r_{2}\cos(\theta -\phi )}{\sqrt{{\left(r_{1}\cos\phi -r_{2}\cos\theta \right)}^{2}+{\left(r_{2}\sin\theta -r_{1}\sin\phi \right)}^{2}+d^{2}}}d\theta^{\;}$$

where, *N*_1_ and *N*_2_ are the turns of two coils, respectively, *d* is the axial distance between coils, *r*_1_, *r*_2_ are the radius of the coils, *θ* and *φ* are the integral factors of the coils which represent angles between the normal of coil-infinitesimal-element and x-axis.

In practical design of the proposed WPT system using resonant inductor in class E power amplifier, the output power *P*_0_, the transmission distance *d*_23_ and the angular frequency *ω* will be given. According to the required power and frequency, the class E power amplifier can be designed. Combining reference [17] and Formula (16), the parameters of resonant inductor *L*_1_ can be obtained.

(16) $$L=\frac{\pi \mu_{rc}\mu_{0}r^{2}N^{2}}{l_{c}+0.9r}$$

where, *μ_rc_* is the relative permeability of the magnetic core, *l_c_* is the core length, *l_c_ = Na + (N − 1)s, s* is the turn spacing.

To analyze the transmission efficiency of this system, we select *N, r* as basic design variable, the function *R (N, r)* of the equivalent input resistance can be obtained. Then, the output power *P*_0_ and the system efficiency *η* can be expressed as functions *P*_0_ *(N, r)*, *η (N, r)*. As a result, the relationship between the coil-parameters *N*, *r* and the system transmission efficiency can be analyzed. The power and efficiency are as follows:

(17) $$\left\{ \begin{array}{l} P_{0}=P_{oc}\\ \eta =\eta_{{}_{E}}\cdot \eta_{c} \end{array}\right.$$

## 3. System Design

### 3.1. Parameter Design Goals of WPT System

**Figure 4:**
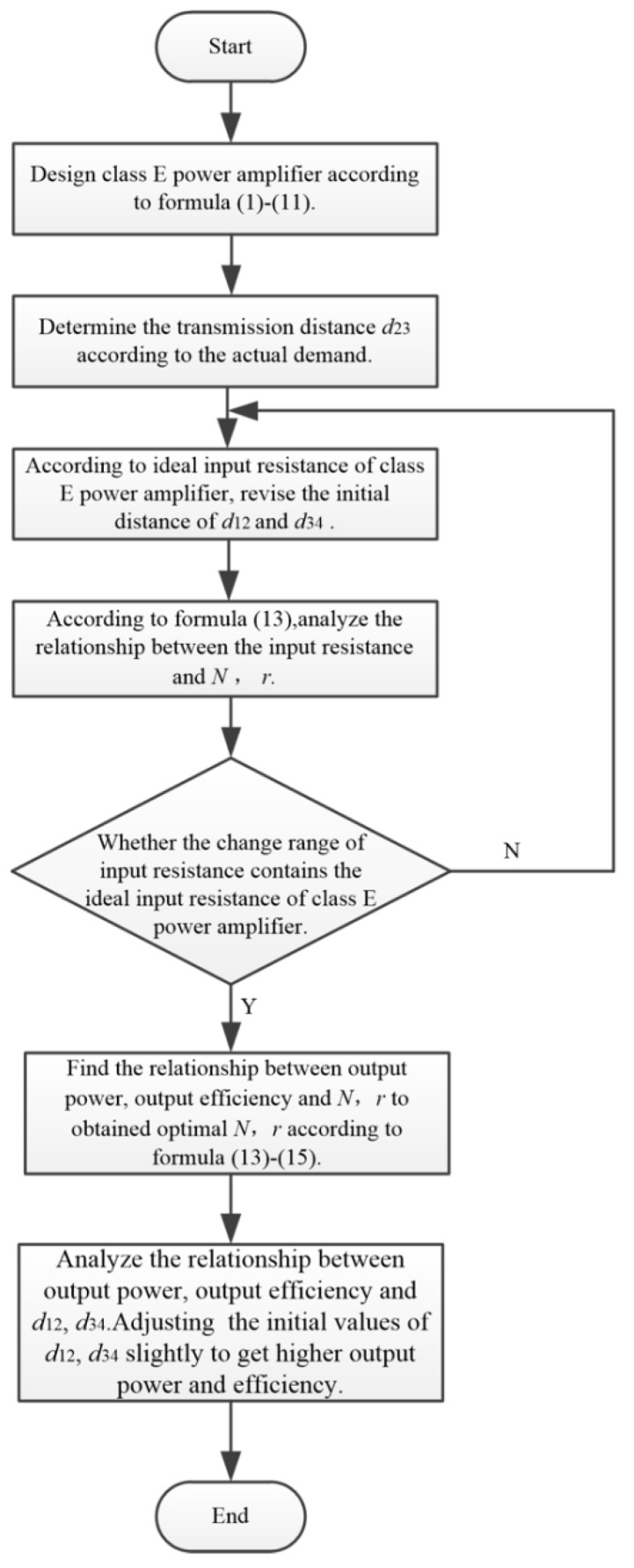
Flow chart of design progress.

First, we presented a flow chart of the design progress in Figure 4.

Where, *d*_12_ is the distance between excitation and transmitter coil. *d*_23_ is the distance between transmitter and receiver coil. *d*_34_ is the distance between receiver and load coil. *N* is the turns of transmitter and receiver coil. r is the radius of all coaxial spiral coils.

In this section, the performance of the proposed WPT system is analyzed by numerical methods. The system design goals are shown in Table 1.

### 3.2. Parameter Design of class E Power Amplifier

Irf200b211 is selected as the switch component of class E power amplifier. According to (8)–(16) and design goals listed in Table 1, the parameters of class E power amplifier can be obtained as shown in Table 2.

**Figure 5:**
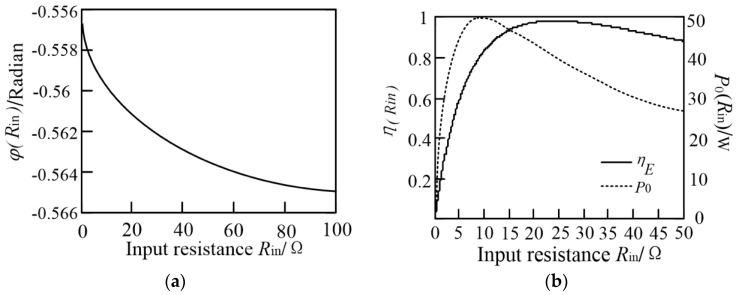
(**a**) Current phase with respect to *R_in_*; (**b**) Efficiency and output power with respect to *R_in_*.

The load current phase, the output power and the transfer efficiency changing with the input resistance *R*_in_ are shown in Figure 5.

It can be concluded that the input resistance *R*_in_ has little effect on the load current phase. When *R*_in_ changes in the range of 1 Ω–100 Ω, the phase is basically remained at around −0.56 radian. The output power and transfer efficiency change obviously with the input resistance *R*_in_ and the maximum value of the efficiency and the power cannot be reached at the same time.

### 3.3. Parameter Design of Coils

The coils designed in this study are all coaxial spiral coils with equal radius *r*. In addition, the turn number *N* of the transmitter and the receiver coil are equal, and the turn number of the load coil is 1. The input resistance of the coils can be adjusted by *d*_12_ and *d*_34_.

**Figure 6:**
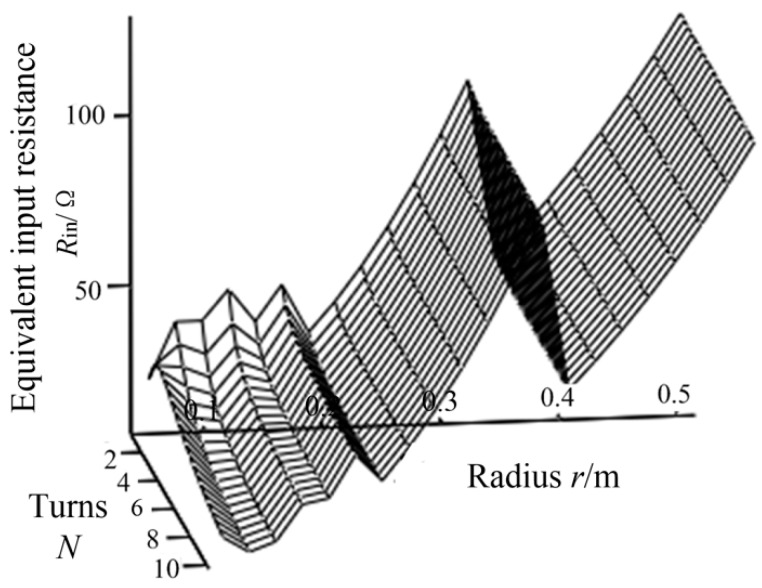
Relationship between the input resistance, *N* and *r*.

According to the design progress shown in Figure 4, *d*_12_ and *d*_34_ need to be adjusted to make *R_in_* contain the ideal value of 23 Ω in the range of variation. In this study, *d*_12_ and *d*_34_ are set to an initial value of 15 cm to meet the requirement of the input resistance. The variation of the input resistance with respect to the turns of transmitter coil (turns of transmitter coil and receiver coil are equal) and coil radius *r* is shown in Figure 6. To facilitate the analysis, the turns of excitation coil is taken as integers, and the inductance value is fine adjusted by turn spacing. When the radius *r* changes, turns of the excitation coil can be obtained according to Formula (18):

(18) $$N=\sqrt{\frac{L_{1}(l_{c}+0.9r)}{\pi \mu_{rc}\mu_{0}r^{2}}}$$

It can be seen from Figure 6 that the equivalent input resistance is in a stepped distribution, which is due to integer turns taken by excitation coil. When the radius *r* increases gradually, in order to ensure that the inductance of excitation coil remains constant, the turns of excitation coil will be stepped down. According to Formula (15), *M*_12_ is directly proportional to turns. The reduction of excitation coil turns will lead to the reduce of mutual inductance *M*_12_. According to Formula (13), the input resistance is directly proportional to the square of *M*_12_, so the input resistance will be greatly reduced when turns of excitation coil is reduced. When the number of the excitation coil turns is constant, the equivalent input resistance increases with the coil radius.

**Figure 7:**
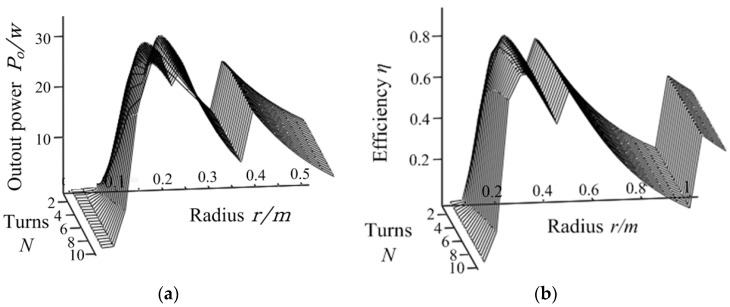
Output power *P*_0_ (**a**) and the efficiency *η* (**b**) with respect to the turns *N* and radius *r*.

**Figure 8:**
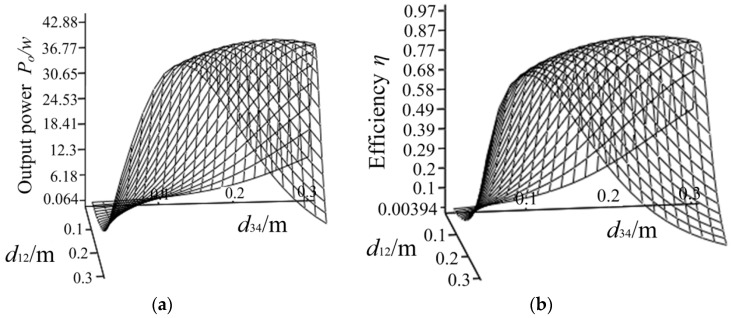
Output power *P*_0_ (**a**) and efficiency *η* (**b**) with respect to *d*_12_ and *d*_34._

According to the analysis above, we can conclude that when the turns of the excitation coil is constant, the equivalent input resistance will increase with the coil radius. Therefore, there is an optimal radius *r* and turns *N* to make the input resistance close to the ideal load resistance (*N* is turns of transmitter and receiver coils, and *r* is radius of each coil.). The variation of power and efficiency with respect to *N* and *r* is shown in Figure 7. It shows that when *N*, *r* change, the output power will be stepped due to the stepped distribution of the input resistance. When the input resistance changes with *N* and *r*, the output power and the efficiency will increase as the input resistance is approaching the optimal load resistance. In addition, due to integer turns taken by excitation coil, a significant reduction of *M*_12_ occurs when radius increases to a certain extent, which makes variation of input resistance and the output voltage. Under the influence of these factors, the efficiency and the power of the system have many peaks and one maximum value. Through the simulation analysis, the optimal turns of the transmitter and receiver coil is *N* = 10, and the optimal radius is *r* = 0.2 m. Although there are peaks of the transfer efficiency and the output power, the maximum efficiency is less than 90%, and the maximum output power is only 31 W when the input power is 38.2 W, which does not reach the ideal working state. This is because we set initial values of *d*_12_ and *d*_34_, the adjustment range of the equivalent input resistance is limited. It is necessary to take *d*_12_ and *d*_34_ into consideration in the progress of the system parameters design. The variation of the system power and efficiency with respect to *d*_12_ and *d*_34_ is shown in Figure 8. There exist optimal values of *d*_12_ and *d*_34_ for this system. The optimal distance is *d*_12_ = 0.16 m and *d*_34_ = 0.17 m. As a result, the system has a maximum efficiency of 93% and an ideal output power of 38 W. The main design parameters of the coil are summarized in Table 3.

## 4. Simulation

### 4.1. Circuit Simulation of the Proposed WPT System and the Traditional Four-Coil WPT System

The parameters of class E power amplifier in the proposed WPT system using resonant inductor are shown in Table 2, and the main design parameters of coils are shown in Table 3. According to these parameters, the WPT system is built and the performance is simulated.

As a contrast, the traditional four-coil WPT system is also set up with the same design goal. The traditional system uses load matching circuit to make the input resistance to ideal state. The class E power amplifier is the same as the proposed type with the parameters in Table 2. By adjusting the distance between the coils and parameters of load matching circuit, the input resistance is matched equal to the ideal input resistance of class E power amplifier. In the traditional type of WPT system, the excitation coil is independent. The excitation and load coils are single-turn.

**Figure 9:**
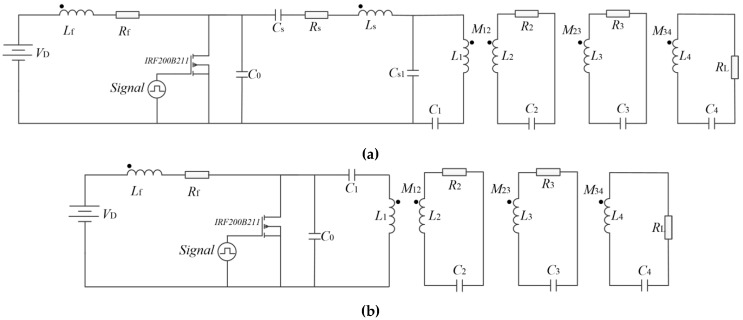
Circuits of (**a**) the traditional four-coil WPT system and (**b**) the proposed WPT system.

According to the design goal, circuit parameters and coil parameters of the traditional four-coil WPT system and the proposed WPT system using resonant inductor can be obtained, which are listed in Table 4 and Table 5. Circuits of the two types of WPT systems are shown in Figure 9.

The simulation results of the proposed system using resonant inductor in class E power amplifier and the traditional four-coil WPT system are shown in Table 6. It can be seen that the performance of the two systems have little difference. This is because the parameters of the two systems are ideal, and the matching circuit can also match the load resistance to ideal state. The coil and tuning capacitor [18] operate at the same frequency, and the equivalent internal resistance of excitation coil is little.

### 4.2. Magnetic Simulation of the Proposed WPT System and the Traditional Four-Coil WPT System

**Figure 10:**
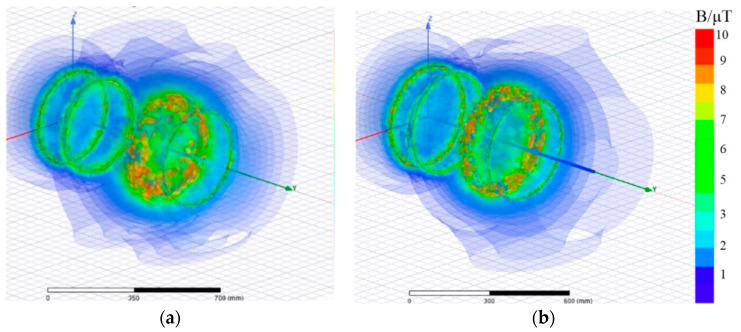
Electromagnetic simulations for magnetic flux B of (**a**) traditional four-coil WPT system and (**b**) proposed WPT system.

Electromagnetic (EM) simulations of the proposed WPT system and the traditional four-coil WPT system were conducted using a EM simulator AnsysEM18.0. The mutual inductance between coils can be calculated by Formula (15). Through Formula (12), the currents in the coils of the WPT system can be obtained. The design parameters of the coils are shown in Table 3 and Table 5. The simulation results are shown in Figure 10.

**Figure 11:**
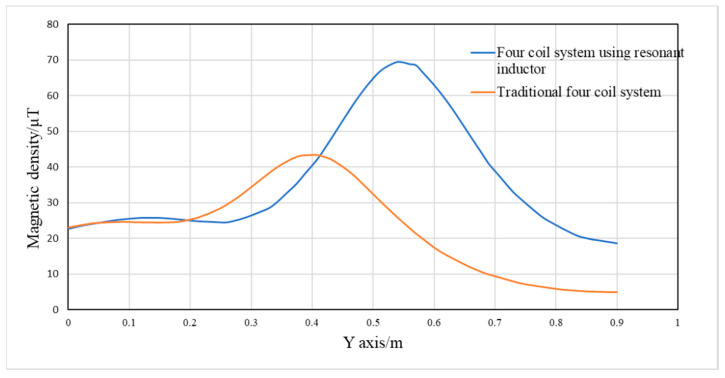
Distribution of magnetic flux density in four-coil system with y-axis direction.

It can be seen from Figure 10 that the magnetic density of the WPT systems is mainly concentrated near the coils. The closer to the coils, the greater the magnetic density will be. The magnetic density of the proposed WPT system is larger. The distribution of the magnetic density of two systems along the y-axis direction is shown in Figure 11. It can be seen from the figure that the magnetic density of the proposed four-coil WPT system using resonant inductor in class E power amplifier is higher than that of traditional four-coil WPT system. In the distance of 0.41 to 0.73 m, the magnetic density of the proposed WPT system is much greater than that of traditional type, which means that the mutual inductance between coils is larger, the transfer efficiency and the output power will be improved.

Table 7 shows the self-inductance and mutual-inductance between the proposed WPT system. It can be seen from the data in the table that the mutual inductance between the coils with a long distance can be ignored except the one between the transmitter coil and the receiver coil due to multiple coil turns. The result is consistent with the theoretical analysis.

## 5. Experiment

**Figure 12:**
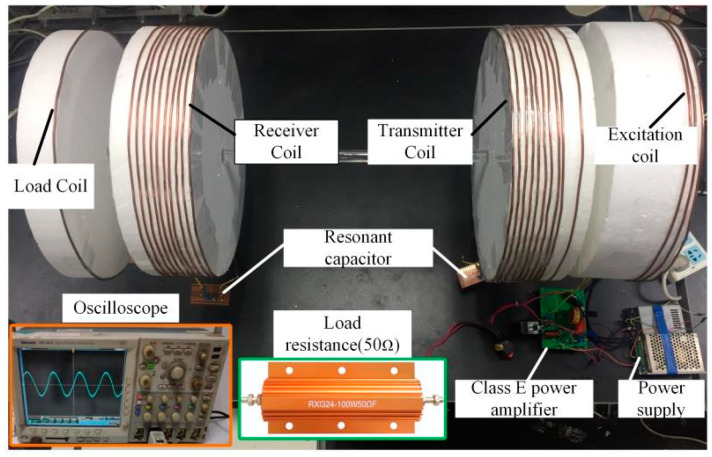
Experimental set up.

The experimental devices are shown in Figure 12. The measuring equipment is Tektronix DPO 4034 digital oscilloscope. The voltage probe is dp6150 high voltage differential probe and the current probe is Tektronix tcp0150. The working frequency of class E power amplifier is 3 MHz, the resonant inductor *L*_1_ = 8.6 μH, the resonant capacitor *C*_1_ = 370 pF, the capacitor in parallel *C*_0_ = 400 pF, the coil radius *r* = 0.2 m, the turns of excitation coil *N*_1_ = 3, the turns of transmitter and receiver coil *N* = 10, and the load coil is single-turn. The distance between the excitation and the transmitter coil is *d*_12_ = 0.15 m, the distance between the transmitter and the receiver coil is *d*_23_ = 0.4 m, and the distance between the receiver and the load coil is *d*_34_ = 0.16 m.

**Figure 13:**
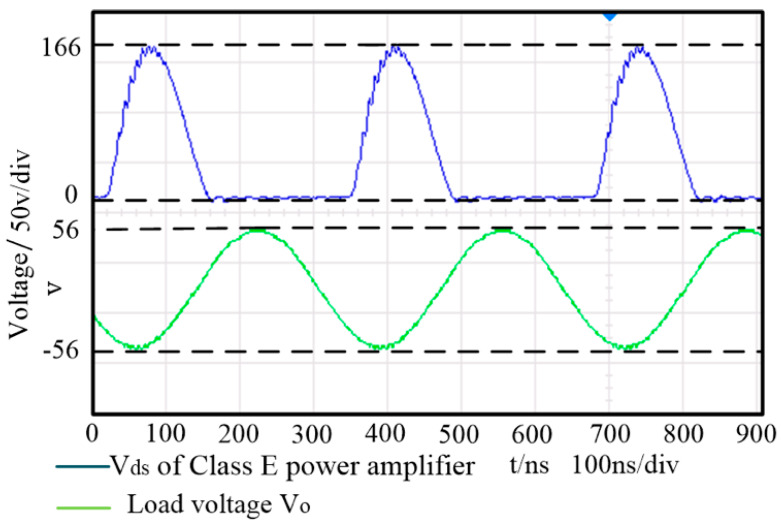
*V_ds_* and load voltage in optimal operating state.

**Figure 14:**
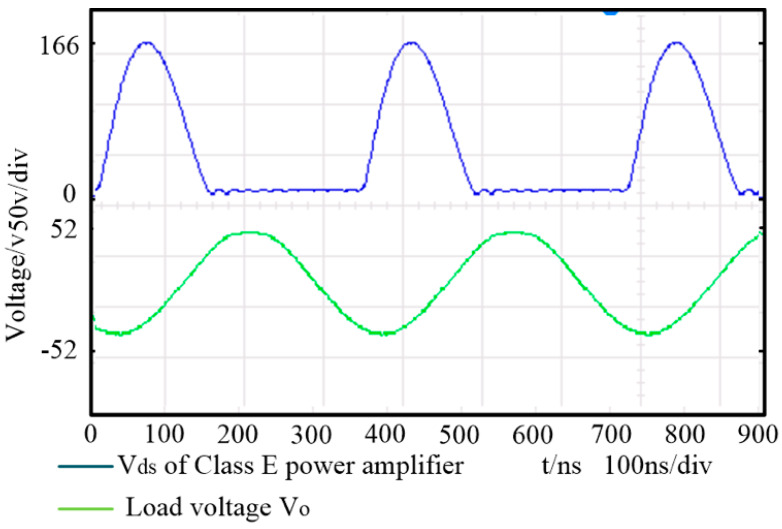
*V_ds_* and load voltage in traditional four coil structure system.

Figure 13 shows the load voltage *V*_0_ and the output voltage of the class E power amplifier *V_ds_* of the proposed WPT system under optimal operating condition. The input current equals 1.1 A. Figure 14 shows *V*_0_ and *V_ds_* of the traditional WPT system under optimal operating condition. The comparison between the experimental results and the theoretical values is shown in Table 8.

It can be seen from Table 8 that the efficiency and output power of the two types of WPT systems are basically consistent with the design index, which verifies the correctness of the proposed design method. According to the *V_ds_* of the class E power amplifier in Figure 13 and Figure 14, it can be seen that the power module works under Zero Voltage Switch (ZVS) and Zero Current Switch (ZCS) conditions with little amplitude of vibration. There are measurement and debugging errors in coil tuning and equivalent input resistance matching, which results in lower efficiency and output power of experimental results than the theoretical results. In the experiment, the input power of the two WPT systems is 44 W. The output power of the proposed WPT system is 31.8 W. Compared with the traditional one, the output power of the proposed WPT system is increased by 18.7% and the transfer efficiency is increased by 11%.

## 6. Conclusions

In this study, a design method of using resonant inductor in class E power amplifier as excitation coil in four-coil WPT system is proposed. In the design process, the mutual inductance between coils and the working state of class E power amplifier under the condition of variable load resistance are analyzed. The design of coupled coils is combined with the class E power amplifier. Under the given design goals, the efficiency and power of the system are expressed as two functions only related to coil turns and radius. Through simulations and experiments, the feasibility of the proposed WPT system is verified.

Compared with the traditional four-coil WPT system, the proposed one has two advantages:

1. The proposed WPT system shares the resonant inductor in class E power amplifier as one resonant coil and does not need the load matching circuit. As a result, the circuit topology and components are simplified. Thus, the reduction of devices, materials and cost are achieved, higher power and efficiency are also guaranteed. The experimental results show that the output power is increased by 18.7% and the transfer efficiency is increased by 11%.
2. The proposed WPT system can work in an ideal condition without precisely tuning of the resonant inductor, which reduces the difficulty of system debugging and improves the stability of the system.
3. The transmission distance of the proposed WPT system reaches 0.7 m, which is much longer than the traditional type. The proposed WPT system is suitable for wireless power supply of electronics and sensors spread across a geographical area.

The results of this study can be used to guide the specific design of long-range WPT system. The present conclusion is derived at a fixed transmission distance *d*_23_. In further study, the design methods will be improved to deal with the changes in the transfer distance and the differences exist between the transmitter coil and receiver coil. WPT system with extremely long transmission range and asymmetrical coils will be developed in practical applications.

## Figures and Tables

**Table 1:** System design goals of WPT system.

Design Goal	Parameters	Unit
Working frequency *f*	3	MHZ
Transmission distance *d*_23_	0.4	m
Output power *P*_0_	40	W
Efficiency *η*	90%	–
Load *R_L_*	50	Ω

**Table 2:** Class E power amplifier parameters.

Design Goal	Parameters	Unit
Resonant inductor *L*_1_	8.56	μH
Resonant capacitor *C*_1_	393	pF
Capacitor in parallel *C*_0_	422	pF
DC voltage *V_D_*	40	V
Ideal load *R_opt_*	23	Ω

**Table 3:** Main design parameters of the coils.

Design Goal	Parameter	Unit
Turns of transmitter and receiver coil *N*	10	turn
Radius *r*	0.2	m
Turns of excitation coil *N*_1_	3	turn
*d*_12_	0.16	m
*d*_34_	0.17	m
Transmission distance *d*_23_	0.4	m

**Table 4:** Circuit parameters of the two types of WPT systems.

Design Goal	Traditional Four-Coil System	Proposed System Using Resonant Inductor	Unit
DC voltage *V*_D_	40	40	V
Choke inductor *L_f_*	55	55	μH
Resistance of choke inductor *R_f_*	0.1	0.1	Ω
Capacitor *C*_0_	360	422	pF
Capacitor *C*_s_	482	/	pF
Capacitor *C*_s1_	1146	/	pF
Resonant inductor *L*_s_	6.7	/	μH
Parasitic resistance *R*_s_	0.1	/	Ω
Inductance of excitation coil *L*_1_	0.89	8.56	μH
Matching Capacitor *C*_1_	3.16	393	pF
Inductance of transmitter coil *L*_2_	64	64	μH
Matching Capacitor *C*_2_	44	44	pF
Resistance of transmitter coil *R*_2_	0.5	0.5	Ω
Inductance of receiver coil *L*_3_	64	64	μH
Matching Capacitor *C*_3_	44	44	pF
Resistance of receiver coil *R*_3_	0.5	0.5	Ω
Inductance of load coil *L*_4_	0.89	0.89	μH
Matching Capacitor *C*_4_	3.16	3.16	nF
Resistance of load *R_L_*	50	50	Ω
Ideal load *R*_opt_	23	23	Ω
Mutual-inductance *M*_12_	1.94	4.00	μH
Mutual-inductance *M*_23_	5.05	2.83	μH
Mutual-inductance *M*_34_	2.03	1.23	μH

**Table 5:** Coil parameters of the two types of WPT systems.

Design Goal	Traditional Four-Coil System	Proposed System Using Resonant Inductor	Unit
Transmitter and receiver coil turn *N*	10	10	turn
Coil radius *r*	0.2	0.2	m
Turns of Excitation coil turn *N*_1_	1	3	turn
*d*_12_	0.115	0.16	m
*d*_34_	0.11	0.17	m
Transmission distance *d*_23_	0.3	0.4	m

**Table 6:** Performance comparison of two types of WPT systems.

Performance Indicator	Traditional Four-Coil System	Proposed System Using Resonant Inductor
DC input voltage	40.0 V	40.0 V
DC input current	1.0 A	1.0 A
Input power	40 W	40 W
Load voltage	42.6 V	43.5 V
Output power	36.2 W	38 W
Efficiency	91%	93%

**Table 7:** Self- and mutual- inductance between coils.

	*L*_1_ (μH)	*L*_2_ (μH)	*L*_3_ (μH)	*L*_4_ (μH)
***L*_1_** (**μH**)	11.034	5.1949	0.51921	0.025264
***L*_2_** (**μH**)	5.1949	88.153	3.2131	0.14413
***L*_3_** (**μH**)	0.51921	3.2131	87.684	1.3048
***L*_4_** (**μH**)	0.025264	0.14413	1.3048	1.628

**Table 8:** Comparison of two types of WPT systems.

Performance Indicator	Theoretical Value of Proposed Type	Experimental Value of Proposed Type	Theoretical Value of Traditional Type	Experimental Value of Traditional Type
DC input voltage	40.0 V	40.0 V	40.0 V	40.0 V
DC input current	1.0 A	1.1 A	1.0 A	1.1 A
Input power	40 W	44 W	40 W	44 W
Load voltage	43.5 V	39.9 V	42.6 V	36.6 V
Output power	38 W	31.8 W	36.2 W	26.8 W
Efficiency	93%	72%	91%	61%
